# Elucidation of the Reinforcing Spleen Effect of Jujube Fruits Based on Metabolomics and Intestinal Flora Analysis

**DOI:** 10.3389/fcimb.2022.847828

**Published:** 2022-03-24

**Authors:** Yan-ling Yi, Yao Li, Sheng Guo, Hui Yan, Xin-fei Ma, Wei-wei Tao, Er-xin Shang, Yang Niu, Da-wei Qian, Jin-ao Duan

**Affiliations:** ^1^ Jiangsu Collaborative Innovation Center of Chinese Medicinal Resources Industrialization, State Administration of Traditional Chinese Medicine Key Laboratory of Chinese Medicinal Resources Recycling Utilization, Nanjing University of Chinese Medicine, Nanjing, China; ^2^ Key Laboratory of Hui Ethnic Medicine Modernization, Ministry of Education, Ningxia Medical University, Yinchuan, China

**Keywords:** *Zizihpus jujuba*, spleen deficiency, metabolomics, intestinal flora, UHPLC–QTOF/MS

## Abstract

Jujube (*Ziziphus jujuba* Mill.) fruit (JF) is widely consumed as food in Asian countries due to its potential effects for human health. As a traditional Chinese medicine, JF is often used to treat anorexia, fatigue and loose stools caused by spleen deficiency syndromes in China, but the mechanism underlying this effect has not been thoroughly elucidated. In this study, a rat model of spleen deficiency syndromes was adopted to investigate the therapeutic effect of JF extract and its possible mechanism by metabolomics analyses of plasma and urine as well as the intestinal flora analysis. The results showed that the changes in plasma and urine metabolites caused by spleen deficiency were reversed after administration of JF, and these changed endogenous metabolites were mainly involved in retinol metabolism, pentose and glucuronate interconversions, nicotinate and niacinamide metabolism pathways. The 16S rDNA sequencing results showed that JF could regulate intestinal flora imbalance caused by spleen deficiency. The covariance analysis of intestinal flora structure and metabolome indicated that *Aerococcus* may be a candidate strain for predicting and treating the metabolic pathways of spleen deficiency and related disorders. In summary, it can be revealed that spleen deficiency, which alters metabolic profiles and the intestinal flora, could be alleviated effectively by JF extract.

## Introduction

Spleen deficiency is a typical clinical symptom in traditional Chinese medicine (TCM), mainly referring to various diseases caused by deficiency or imbalance of Yin and Yang, Qi and Xue of the spleen (Wang et al., 2020). In the theory of TCM, “spleen” is completely different from the spleen organ in Western medicine. The spleen is a comprehensive structural and functional concept that includes not only the spleen in modern anatomy, but also the stomach, pancreas and lymphatic system (Zhang et al., 2016). Spleen deficiency syndrome is clinically manifested by loss of appetite, indigestion, fullness and drowsiness after eating, fatigue, white face and tongue, weight loss, abdominal distension, loose stools or diarrhea, etc., which is a common subhealth state described by Chinese medicine (Lin et al., 2018; Ma et al., 2019; Peng et al., 2020; Shi et al., 2020). The occurrence and development of spleen deficiency will also be closely related to gastrointestinal digestive dysfunction, intestinal inflammatory lesions, low immune status (Wang et al., 2015; Xue et al., 2018), and water metabolism disorders (Cao et al., 2018; Camilleri et al., 2019). Interestingly, in TCM, most patients with cancer cachexia were diagnosed with spleen deficiency (Zhang et al., 2020c). Spleen deficiency has developed into an increasingly non-negligible health problem. Although spleen deficiency is not life-threatening, it seriously affects the patient’s health and quality of life. The research on spleen deficiency has received increasing attention from experts and scholars.

Jujube fruit (JF), the mature fruit of *Ziziphus jujuba* Mill. (Rhamnaceae) is widely consumed as food in Asian countries due to its potential effects for human health. It has also been used as a traditional herbal medicine for thousands of years. According to the earliest Chinese medical monograph, *Shennong Ben Cao Jing* (300 BC−200 AD), JF was considered one of the excellent herbal medicines (Chen and Tsim, 2020). TCM believes that JF has the effect of reinforcing spleen and stomach, and is commonly used for the treatment of anorexia, fatigue and loose stools related to deficiency syndromes of the spleen and of hysteria in woman (Gao et al., 2013). Phytochemical studies have shown that JF contains a variety of chemical constituents, including triterpene acids, flavonoids, saponins, alkaloids, phenolic acids, polysaccharides, amino acids, and other components (Guo et al., 2013; Guo et al., 2015; Bai et al., 2016). Although JF has been used for thousands of years to nourish the spleen and benefit the stomach, and modern pharmacological studies have confirmed the efficacy of JF, its overall effect on the body and the biological mechanism have not been elucidated.

Due to the diversity of chemical components of JF and the complexity of drug-organism interactions, it is not feasible to reveal the multi-component synergistic and multi-target characteristics of herbal medicines using only a single index or a simple superposition method (Jin et al., 2016). Metabolomics is a holistic approach to the analysis of endogenous low-molecular-weight metabolites in various biological samples, which allows simultaneous monitoring of multiple metabolic pathways and reasonable speculation on the regulation of these pathways based on metabolite expression (up- or down- regulation) data (Zhang et al., 2020a). This concept is consistent with the integral and systematic characteristics of TCM theory, which focuses on harmonizing the overall internal and external environment of the human body, maintaining the balance of physiological functions of the body, and then improving its immunity and resistance (Li et al., 2015; Wang et al., 2018). Therefore, metabolomics has been widely used in efficacy evaluation and clarification of possible mechanisms of TCM (Yan et al., 2020; Yang and He, 2020). Meanwhile, the application of metabolomics to animal models of spleen deficiency has been reported in the literatures (Liu et al., 2020; Huang et al., 2020).

Notably, the balance of intestinal flora is crucial to human health, and it plays an important role in maintaining normal intestinal functions, including peristalsis, digestion, absorption, barrier, metabolism, and immunity (Xu et al., 2017). More and more studies have shown that herbal medicine can exert therapeutic effects by regulating intestinal flora metabolism, such as kidney Yang deficiency syndrome, acute liver injury, colon cancer, hypertension, etc. (Chen et al., 2019; Liu et al., 2019; Ji et al., 2020; Qi et al., 2020). Spleen deficiency is also considered to be closely related to the imbalance of intestinal flora (You et al., 2020). Therefore, exploring and revealing the relationship between metabolomics and structural alterations of intestinal flora of spleen deficiency can not only enrich and improve the essence of spleen deficiency, but also provide means to discover drugs for treating spleen deficiency and clarify its molecular mechanism.

For the above purpose, UHPLC-Q-TOF/MS was used in this study to analyze the plasma and urine metabolomics of spleen-deficiency rats after JF intervention, and 16S rDNA sequencing was used to analyze the composition of their intestinal flora. Furthermore, covariance analysis of metabolites with specific intestinal flora profiles was performed to reveal potential links between changes in host metabolome and intestinal flora structure. Through the above research, this study aimed to elucidate the potential mechanism of JF for tonifying spleen and stomach from the perspective of metabolomics and intestinal flora, which can also provide reference for relevant research.

## Materials and Methods

### Materials

JF was collected in Cangzhou, Hebei Province and *Sennae Folium* were collected in Hainan Province, China. The herbs were identified as the dried fruits of *Ziziphus jujuba* Mill. and dried leaves of *Cassia acutifolia* Delile, respectively, by professor Jin-ao Duan, Nanjing University of Chinese Medicine. After collection, the JF (JF-20161018) and *Sennae Folium* (SF-20160923) voucher specimens were deposited in the Jiangsu Collaborative Innovation Center of Chinese Medicinal Resources Industrialization, Nanjing University of Chinese Medicine under closed and dry conditions at 25 ± 5°C. Shen-Ling-Bai-Zhu granule (SL) was purchased from the Beijing Tong Ren Tang Pharmaceutical Co., Ltd.

Acetonitrile and formic acid were both obtained from Merck KGaA (Darmstadt, Germany). Methanol was purchased from TEDIA Company Inc. (Fairfield, USA). D-xylose was purchased from Aladdin reagent Co., Ltd. (Shanghai, China). Deionized water was purified by a Milli-Q system (Millipore, Bedford, USA).

### Preparation of Extract

*Sennae Folium* (1.5 *kg*) was refluxed with 15 L water for extraction twice, 30 min each time. The extract was combined after filtration, and the solvent was concentrated at 60°C to obtain the decoction of *Sennae Folium* containing 1 g of raw drug per ml. JF (1 *kg*) was cut in half and extracted twice with 6 L water at reflux for 3 h each time. The filtrate was combined and the solvent was recovered to obtain JF decoction with crude drug content of 1.35 g/ml. The extracts were stored at -30°C and diluted with pure water to the required dose when needed. The positive control drug Shen-Ling-Bai-Zhu granule was dissolved in distilled water to prepare the solution containing 0.56 g granule per ml.

### Animals

Male Sprague-Dawley rats (200 ± 20 g) were provided by Zhaoyan New Drug Research Center Co., Ltd (Suzhou, China). Permit number: SCXK (SU) 2013-0003. Animals were housed in Drug Safety Evaluation Center of Nanjing University of Chinese Medicine, Nanjing, China. Animal welfare and all protocols of the study were approved by the Animal Ethics Committee of Nanjing University of Chinese Medicine. These rats received food and water ad libitum.

After 7 days of acclimatization, the rats were randomly divided into 6 groups with 6 rats in each: control group (C); model group (M); positive control group (SL, orally administered Shen-Ling-Bai-Zhu granule with the dose of 5.6 g/kg); low dose JF group (JFL, orally administered JF extract with the dose of 2.7 g crude herbs per 1 kg rat weight), medium dose JF group (JFM, orally administered JF extract with the dose of 6.75 g crude herbs per 1 kg rat weight) and high dose JF group (JFH, orally administered JF extract with the dose of 13.5 g crude herbs per 1 kg rat weight). Except for the control group, the other rats were given *Sennae Folium* extract by intragastric administration at a dose of 10 g/kg for continuous 10 days. During the 10-day modeling process, the rats in each treatment group were given Shen-Ling-Bai-Zhu granule or JF by oral administration. The rats in the control group and model group were given the same amount of water by gavage. The administered volume was 10 ml/kg for each rat, each time. The animals’ weight was recorded every two days.

### Sample Collection and Determination of Biochemical Indicators

After the last administration, fresh feces of rats were collected in a sterile centrifuge tube and stored in a refrigerator at -80°C for further determination of intestinal flora. On the 10th day of gavage, the rats were fasted in metabolic cages, and 12 h urine samples were collected. All samples were immediately centrifuged at 1000 *×g* for 10 min after collection. Then, the supernatant was separated and stored at -80°C until analysis. After gavage of 5% D-xylose solution for 2 hours on the 11th day, 10% choral hydrate (350 mg/kg, intraperitoneal injection) was used for anesthesia. Blood samples (4 ml) of rats were collected by abdominal aortic intubation approach into 5 ml blood sample collection container with EDTA.K_2_. Then, 800 µl of whole blood were used to measure peripheral blood routine which mainly includes white blood cell count (WBC, 10^9^/l), red blood cell count (RBC, 10^12^/l), lymphocyte ratio (LYMPH, %), monocytes ratio (MONO, %), neutrophils ratio (GR, %) and platelet count (PLT, 10^9^/l) by ADVIA120 type fully automatic blood analyzer (Bayer, Germany). The rest of the whole blood samples were centrifuged at 1000 *×g* for 10 min. The supernatant of plasma samples was separated and stored at -80°C until UHPLC-QTOF/MS analysis. The rest of the rat blood samples was collected into a blood sample collection container without EDTA.K2. The blood samples were immediately centrifuged at 1000 *×g* for 10 min. The supernatant of serum samples was separated and stored at -80°C for further determining the contents of gastrin and somatostatin, and the activities of cholinesterase and amylase by using enzyme-linked immunosorbent assay (ELISA). All ELISA kits were purchased from Nanjing Jiancheng Bioengineering Institute (Jiangsu, China), and each step of the experiment was strictly in accordance with the kit instructions. The content of D-xylose in serum was determined with the commercially available assay kits (Nanjing Jiancheng Bioengineering Institute, China). The spleen and small intestine tissues were quickly removed. The spleen was stripped and weighed, and the spleen index was calculated according to the following formula: spleen index = spleen weight/body weight (g/kg). The small intestine tissues were stored in 4% paraformaldehyde for pathological analysis.

### Histopathological Analysis

The small intestine tissues fixed with 4% paraformaldehyde solution were dehydrated, embedded in paraffin, and cut into 4-μm-thick slices. The slides were stained with hematoxylin and eosin (H&E), then observed under light microscopy at 200× using an optical microscope.

### Metabolomics Study on Plasma and Urine

Plasma and urine samples were thawed at room temperature before preparation. Methanol (300 µl) was added into each plasma (100 µl) and urine sample (100 µl) to precipitate protein. Afterwards, the mixture was vortexed for 60 s and centrifuged at 16060 *×g* for 15 min. Then, 200 µl supernatant of the plasma and urine samples was used for UHPLC-MS analysis. Besides, the quality control (QC) sample was prepared to monitor the reproducibility and stability of the acquisition system. The plasma (or urine) samples were randomly selected from the control and model groups and mixed together as the QC samples, respectively. The QC samples were analyzed every nine samples throughout the whole analytical run.

Experiments was carried out on a Waters Acquity UPLC™ system (Waters Corp., Milford, MA, USA) using a Grace VisionHT C_18_ column (100 mm × 2.0 mm, 1.5 µm). The column temperature was 35°C during the analysis. The mobile phase was composed of 0.1% formic acid solution (A) and acetonitrile (B) at a flow rate of 0.4 ml/min. The gradient eluting conditions for plasma samples were as follows: 0-3 min, 95%-55% A; 3-13 min, 55%-5% A; 13-14 min, 5% A; the UHPLC elution conditions for urine samples were as follows: 0-8 min, 95%-70% A; 8-11 min, 70%-30% A; 11-13 min, 30%-5% A; 13-14 min, 5% A. The 2 µl sample volume was injected for analysis.

Mass spectrometry was accomplished on a Waters Synapt™ QTOF/MS (Waters Corp., Milford, MA, USA) equipped with an electrospray ionization (ESI) source in both positive and negative ion mode. The parameters of ionization source were set as follows: source temperature of 120°C, capillary voltage of 3 kV, desolvation temperature of 350°C, sampling cone voltage of 30 V, extraction cone voltage of 2 V. The desolvation and cone gas were both nitrogen, and set respectively at the flow rate of 600 and 50 L/h. The scan range was between m/z 100 to 1000 Da. The scan time, interval scan time and collision energy were set respectively at 0.5 s, 0.02 s and 6 eV throughout the whole experiments. Leucine-enkephalin was used as the lock mass which is generating an [M + H] ^+^ ion (m/z 556.2771) and [M − H] ^−^ ions (m/z 555.2615) in positive and negative modes, respectively. The concentration of Leucine-enkephalin was set at 200 pg/ml and the infusion flow rate was 50 μl/min.

The obtained UHPLC-QTOF/MS data of all samples were analyzed by Masslynx software (version 4.1, Waters Corp. Milford, MA, USA) for peak detection and alignment. For data collection, the method parameters were set as that: retention time range 1.0-14.0 min, mass range 100-1000 Da, mass tolerance 0.05 Da, and noise elimination level 20. Then EZinfo 2.0 software was used to analyze the resulting data by its principal component analysis (PCA), partial least squares discriminant analysis (PLS-DA) and orthogonal projection to latent structures (OPLS) analysis. All variables obtained from UHPLC-MS data sets were mean-centered and scaled to pareto variance before using PCA, PLS-DA and OPLS-DA. The quality of the model was described by the cross-validation parameter Q^2^ and R^2^Y of PLS-DA score plots, which indicated the predictability of the model and the total explained variation for the X matrix, respectively. In this study, the variables with variable importance in the projection (VIP) > 1 were considered to be influential for the separation of samples in the score plots generated from PLS-DA analysis (Lenz and Wilson, 2007). Once potential biomarker candidates had been selected from the data analysis, they were mainly identified by online HMDB. Pathway analysis was performed with MetaboAnalyst 3.0.

### Gut Flora Analysis

Feces samples were used to extract DNA using the MoBio PowerSoil^®^ DNA Isolation Kit (12888). The extracted DNA was determined by molecular size using 0.8% agarose gel electrophoresis and quantified using UV spectrophotometry. The V4 highly variable region of 16S rDNA was amplified, and the pre-amplified primer sequence was the specific primer 520F (5’-tag +GCACCTAAYTGGGYDTAAAGNG-3’), and the post-amplified primer sequence was 802R (5’-TACNVGGGTATCTAATCC-3’). The PCR amplification products were detected by electrophoresis using 2% agarose gel; the PCR products were mixed in equal concentrations according to the PCR products, and the PCR products were detected by electrophoresis using 2% agarose gel after sufficient mixing, and the target fragments were cut and recovered. PCR products were mixed quantitatively and then used to construct gene libraries using Illumina’s TruSeq Nano DNA LT Library Prep Kit. Finally, 2 × 300 bp double-end sequencing was performed with MiSeq Reagent Kit V4 (600 cycles). The raw data after sequencing were double-ended spliced using FLASH 1.2.7 software (http://ccb.jhu.edu/software/FLASH/). Finally, according to the Index information (i.e. Barcode sequence) corresponding to each sample, the concatenated sequences were identified and assigned to the corresponding samples (exact match of Index sequences is required), thus obtaining the valid sequence of each sample. During the high-throughput sequencing process, a series of erroneous or questionable sequences may be generated. In order to ensure reliable and accurate analysis results, QIIME software was applied to the valid sequences obtained by the above extraction to identify the questionable sequences, and finally USEARCH was called by QIIME software to check and reject the chimeric sequences, so as to obtain high-quality sequences. High-quality sequences with similarity higher than 97% were grouped into an Operational Taxonomic Unit (OTU) using UCLUST software, and OTUs with abundance values lower than 0.001% of the total sequencing of all samples were removed, and sequences with rare OTUs removed were compared with the relevant database Greengenes. The structural composition of the microbial community was obtained by performing abundance, Beta diversity analysis, LEfSe analysis and statistical analysis of the community results of species at each taxonomic level for OTUs.

### Correlation Analysis Between Metabolites and Intestinal Flora

The Pearson correlation coefficient was used to show the relationship between the parameters (linear correlation) and the correlation coefficient was always between -1 and +1. If the absolute value of the correlation coefficient was closer to 1, the better the linear relationship. In this experiment, Pearson correlation coefficients r > 0.6 and r < - 0.6 were used to indicate significant positive and significant negative correlations, respectively.

### Statistical Analysis

Data were expressed as mean ± SD, and SPSS 22.0 statistical software was used for statistical processing. ANOVA analysis of variance was used when the data were normally distributed and the variances were homogeneous for comparisons between multiple groups; otherwise, Kruskal-Wallis test was used.

## Results

### Assessment of Spleen Deficiency Model and Reinforcing Spleen Effect of JF Extract

**Figure 1:**
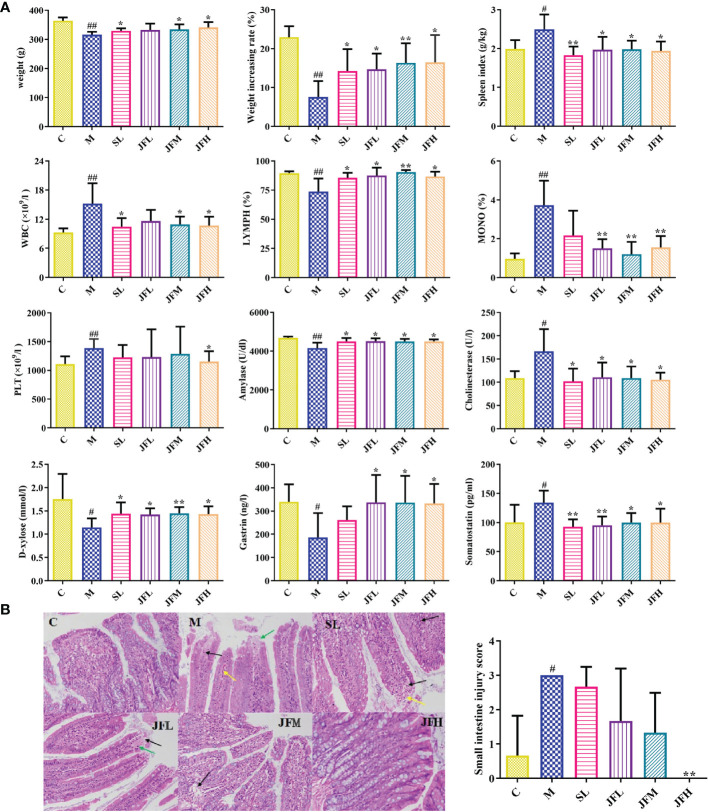
Determination of appearance indicator, peripheral blood routine and biochemical indicators **(A)**; pathological sections of small intestine HE staining **(B)**. WBC: white blood cell count; LYMPH: lymphocyte ratio; MONO: monocytes ratio; PLT: platelet count. (C) control group; (M) model group; (SL) positive group; (JFL) low dose group of JF extract; (JFM) medium dose group of JF extract; (JFH) high dose group of JF extract. Data are expressed as means ± SD, n = 6. ^#^P < 0.05, ^##^P < 0.01: model vs control; *P < 0.05, **P < 0.01: treatment vs model.

The results of the appearance index, peripheral blood routine and biochemical indicators are shown in **Figure 1A**. Compared with the control group, the weight, weight increasing rate, lymphocyte ratio (LYMPH) and the levels of serum amylase, D-xylose and gastrin were significantly decreased in the model group, while spleen index, white blood cell count (WBC), monocyte ratio (MONO), platelet count (PLT) and the levels of serum cholinesterase and somatostatin were significantly increased. Compared with the literatures (Zhao et al., 2020; Chen et al., 2021), the above results indicated that the spleen deficiency model induced by *Sennae Folium* was successful. After the administration of JF, the above indicators tended to recover to the normal levels. HE staining results of small intestine tissues in each group are shown in **Figure 1B**. Microscopic observation showed that there were more eosinophil infiltration in intestinal mucosa lamina propria, and local small-scale necrosis and exfoliation of intestinal mucosa epithelium in model group rats. The tissue injury score was significantly different from that of the blank group. The high-dose JF group (JFH) showed a significant improvement of the injury caused by *Sennae Folium*, which indicated that JF can alleviate the symptoms of spleen deficiency.

### Metabolism Analysis

**Figure 2:**
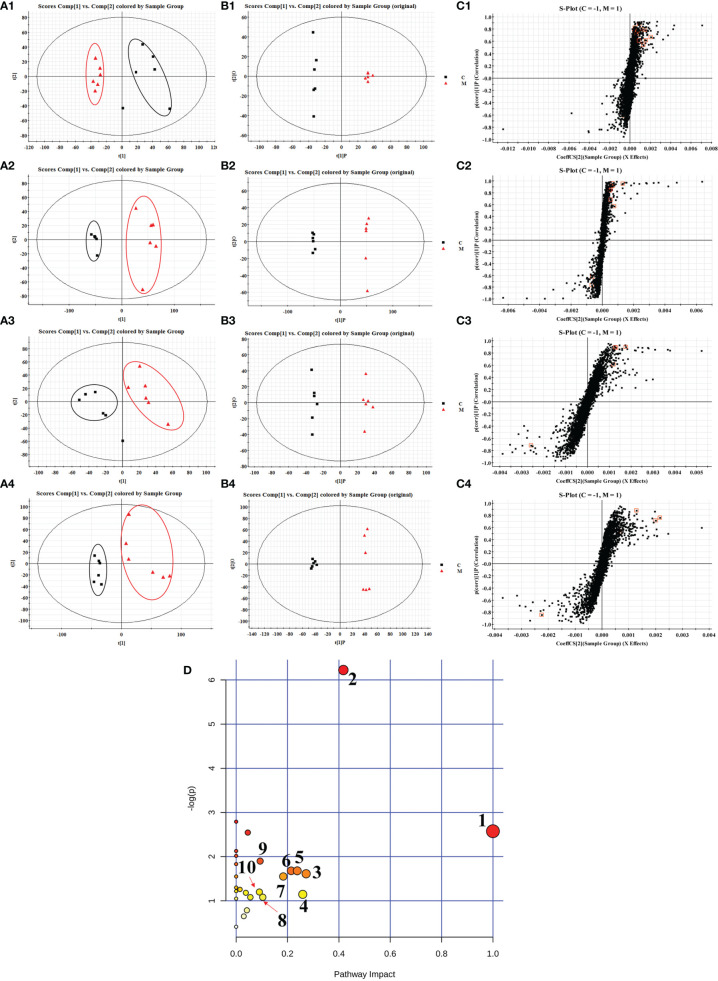
PCA scores plots **(A)**, OPLS-DA scores plots **(B)** and S-plots of OPLS-DA **(C)** for plasma (1, 2) and urine (3, 4) of model group (red) vs. control group (black) in positive (1, 3) and negative (2, 4) ion mode; summary of pathway analysis in plasma and urine with MetPA **(D)**. (1) D-glutamine and D-glutamate metabolism; (2) retinol metabolism; (3) pentose and glucuronate interconversions; (4) alanine, aspartate and glutamate metabolism; (5) nicotinate and niacinamide metabolism; (6) ether lipid metabolism; (7) selenoamino acid metabolism; (8) glycolysis or gluconeogenesis; (9) arginine and proline metabolism; (10) steroid hormone biosynthesis.

The separation and raw data collection of plasma and urine samples were performed by UHPLC-QTOF/MS. In order to explore the overall changes of metabolite groups in the rats of spleen deficiency model group, PCA and OPLS-DA methods were used to perform pattern recognition on the plasma/urine metabolic profile data of the control and model group rats to obtain the sample distribution maps, which could reflect the degree of similarity and difference between the samples. As shown in the PCA score plots (**Figures 2A1–A4**), plasma and urine samples from the model group (M) and the control group (C) showed significant clustering in both positive and negative ion patterns, indicating that metabolic abnormalities occurred in the spleen deficient rats compared to the control group. The potential markers of interest (marked in red boxes) were subsequently extracted from the S-plots (**Figures 2C1–C4**) constructed by OPLS-DA (**Figures 2B1–B4**). The metabolites in plasma and urine samples were identified based on MS/MS data, KEGG, HMDB 3.6 and PubChem, and a total of 31 metabolites were annotated (22 metabolites from plasma and 9 metabolites from urine). The mass spectrometry data and their changing trends in the model group compared to the control group are shown in **Table S1**.

**Figure 3:**
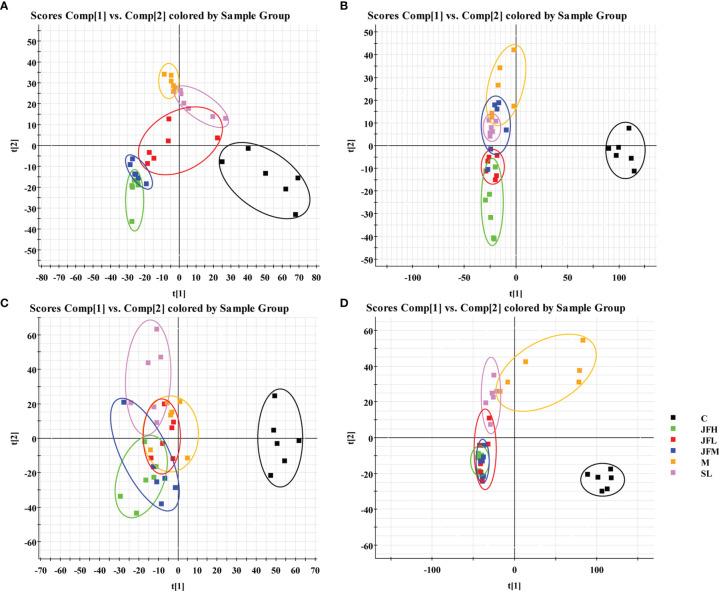
PLS-DA score plots of rat plasma and urine samples classifying the C, M, SL, JFL, JFM and JFH in both positive and negative modes. **(A)** ESI^+^ mode of plasma; **(B)** ESI^-^ mode of plasma; **(C)** ESI^+^ mode of urine; **(D)** ESI^-^ mode of urine.

**Figure 4:**
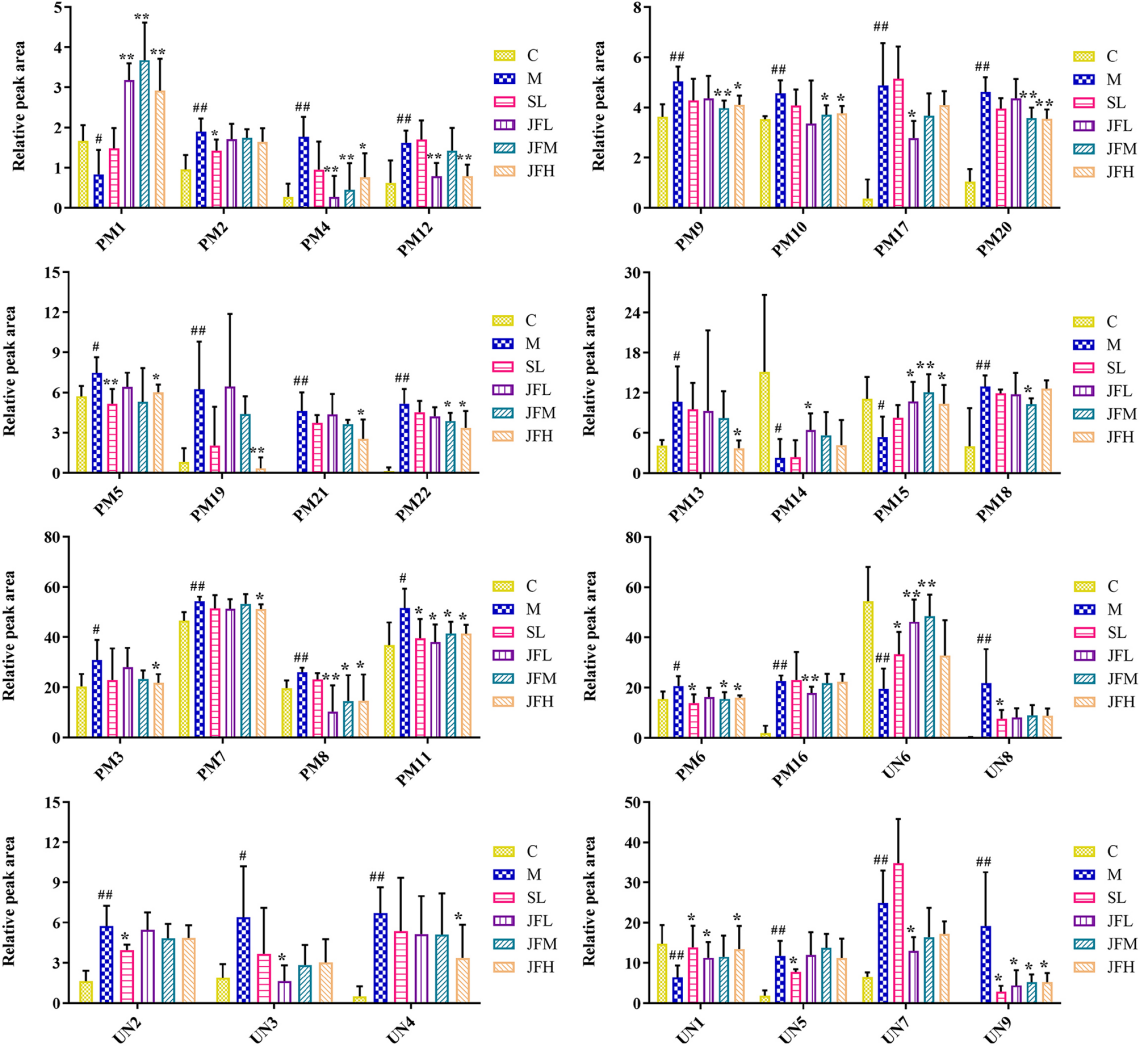
Changes in the relative intensity of target metabolite of plasma and urine identified by UHPLC-QTOF/MS. (C) control group; (M) model group; (SL) positive group; (JFL) low dose group of JF extract; (JFM) medium dose group of JF extract; (JFH) high dose group of JF extract. Data are expressed as means ± SD, n = 6. ^#^P < 0.05, ^##^P < 0.01: models vs control; *P < 0.05, **P < 0.01: treatment vs model.

The UHPLC/Q-TOF/MS data of normal, model, SL, JFL, JFM and JFH groups in positive and negative ion mode were analyzed by PLS-DA analysis to obtain the changes in the control, model and each administration group of rats (**Figure 3**). The relative levels of 31 endogenous metabolites in plasma and urine of rats were statistically examined, and the results (**Figure 4**) showed that 21 endogenous metabolites (except PM2) in plasma and 6 (except UN2, UN5 and UN8) in urine of the JF administration groups were returned to normal levels in different degrees compared with the model group, which indicated that the abnormal metabolism of spleen-deficiency rats was effectively improved.

In order to explore the possible pathways of JF extract on spleen deficiency rats, endogenous metabolites in **Table S1** were imported into MetaboAnalyst 4.0 database (https://www.metaboanalyst.ca/faces/ModuleView.xhtml). The metabolic pathways with effect values greater than 0.1 were selected as the potential target pathways (Wang et al., 2012). Twenty-seven metabolic pathways were constructed, which were important for the host response to the spleen deficiency. As shown in **Figure 2D**, after the intervention of JF, the retinol metabolism, pentose and glucuronate interconversions, nicotinate and niacinamide metabolism, ether lipid metabolism, selenoamino acid metabolism and steroid hormone biosynthesis pathways in spleen deficient rats were improved. Among them, retinol metabolism, pentose and glucuronate interconversions, nicotinate and niacinamide metabolism were filtered out as the most important metabolic pathway for their high impact-value.

### Gut Flora Analysis

**Figure 5:**
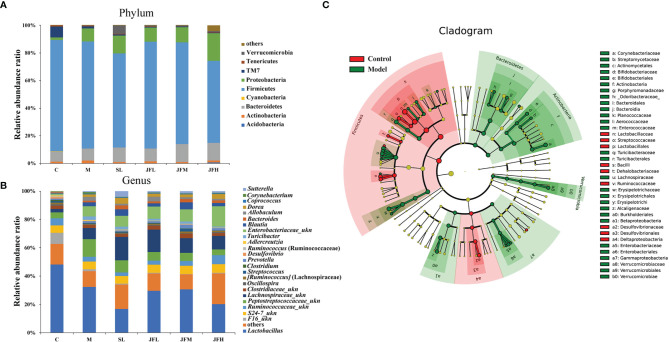
Effects of JF extract on the structure of intestinal flora in spleen-deficiency rats. The percent of community abundance at the phylum **(A)** and genus **(B)** levels. LEfSe hierarchical tree **(C)** of between control group and model group.

The structure and diversity of intestinal flora in rats with spleen deficiency caused by *Sennae Folium* were analyzed by 16S rDNA sequencing, and the regulation effect of JF on the abnormal intestinal flora was investigated. Taxonomic composition analysis of the intestinal flora (**Figures 5A, B**) showed that: at the phylum level, there was no significant difference in the overall structure of the microflora in each group, among which Firmicutes had the highest relative abundance, followed by Proteobacteria and Bacteroidetes; at the genus level, the relative abundance was higher for *Lactobacillus*, followed by *Peptostreptococcaceae_ukn, Lachnospiraceae_ukn and Enterobacteriaceae_ukn.*

**Figure 6:**
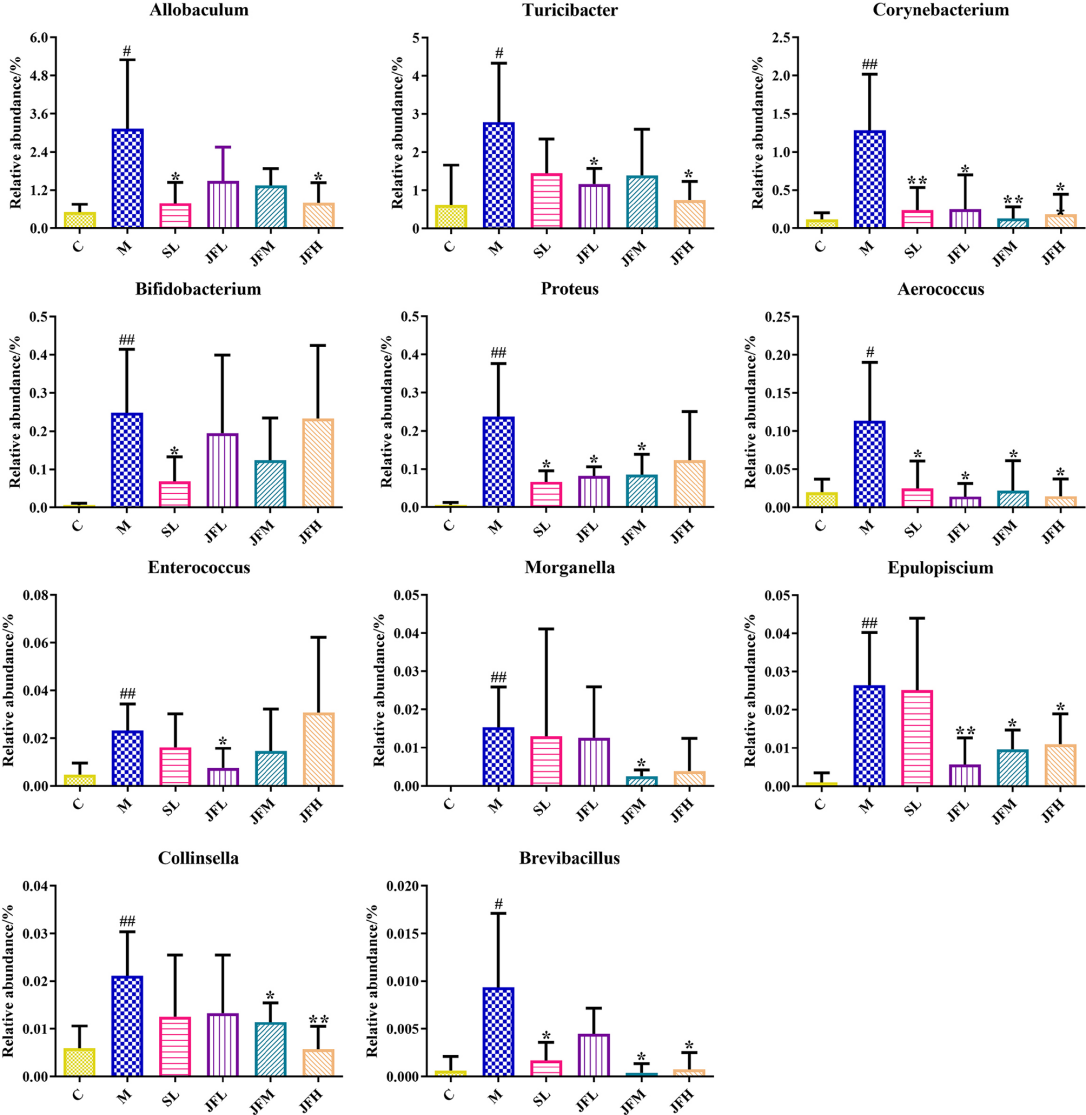
Average relative abundance of difference features flora in feces of all groups. (C) control group; (M) model group; (SL) positive group; (JFL) low dose group of JF extract; (JFM) medium dose group of JF extract; (JFH) high dose group of JF extract. Data are presented as mean ± SD, n = 6. ^#^ *P* < 0.05, ^##^P < 0.01: model vs control; *P < 0.05, **P < 0.01: treatment vs model.

By LEfSe analysis (**Figure 5C**), differential flora was found at the taxonomic levels of phylum, order, family and genus in the feces of rats in spleen deficiency model group compared with the control group. A total of 31 differential genera were found in the genera classification of the bacteriophages, among which *Allobaculum*, *Turicibacter*, *Corynebacterium*, *Sutterella*, *Coprococcus*, *Akkermansia*, *Bifidobacterium*, *Proteus*, *Aerococcus*, *Enterococcus*, *Epulopiscium*, *Morganella*, *Collinsella*, *and Brevibacillus* were upregulated in the model group, while *Psychrobacter*, *Anaerostipes*, *Dehalobacterium*, *Ruminococcus*, *Oscillospira*, *Streptococcus*, *Desulfovibrio*, and *Lactobacillus* showed a down-regulation trend in the model group. JF could improve the relative abundance of *Allobaculum*, *Turicibacter*, *Corynebacterium*, *Proteus*, *Aerococcus*, *Enterococcus*, *Epulopiscium*, *Morganella*, *Collinsella* and *Brevibacillus* in the spleen deficiency model in varying degrees (**Figure 6**).

### Covariation Analysis Between Intestinal Flora Structure and Metabolome

**Figure 7:**
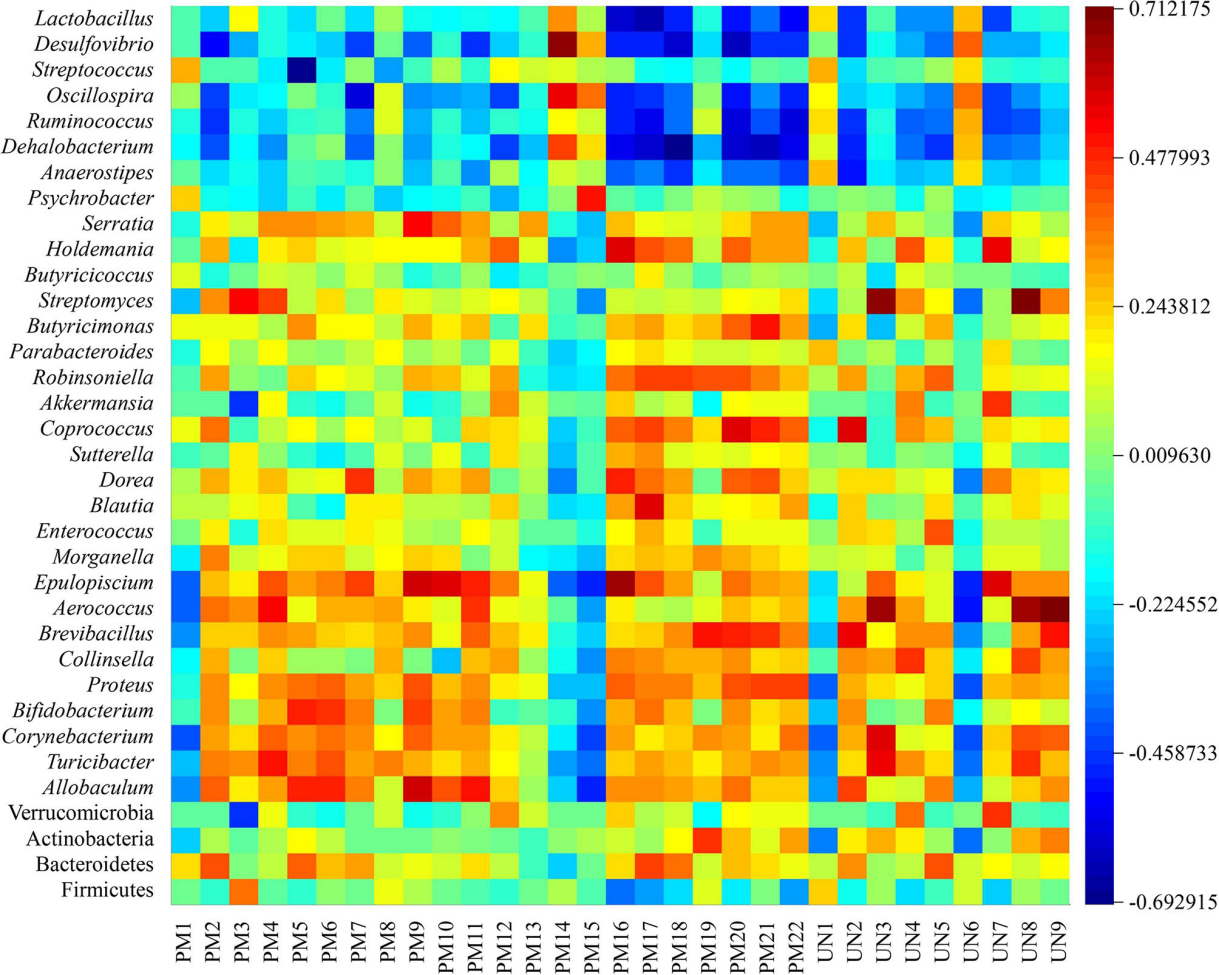
Correlation analysis between potential biomarkers and differential intestinal flora.

To reveal the potential associations between structural changes in the intestinal flora and changes in the host metabolites, Pearson correlation coefficients were calculated for 31 potential biomarkers identified and differential flora (at the phylum and genus levels) to generate correlation matrices. As a result, a map of intestinal flora-host metabolites interactions in the pathological state of spleen deficiency was constructed, as shown in **Figure 7**. A total of four genera were positively correlated with five potential differential metabolites (r > 0.6), including *Desulfovibrio* with taurocholic acid (PM14), *Streptomyces* with niacinamide (UN3) and 17α-hydroxypregnenolone (UN8), *Epulopiscium* with 3-methyl-1-hydroxybutyl-ThPP (PM17), and *Aerococcus* with niacinamide (UN3), 17α-hydroxypregnenolone (UN8) and vitamin A (UN9). Four genera were found to be negatively correlated with five potential biological metabolites (r < -0.6), such as *Lactobacillus* with LysoPC (22:4(7Z,10Z,13Z,16Z)) (PM16), *Desulfovibrio* with 5-amino-6-(5’-phosphoribitylamino) uracil (PM20), *Streptococcus* with LysoPC (22:6(4Z,7Z,10Z,13Z,16Z,19Z)) (PM5), and *Dehalobacterium* with all-trans-hexaprenyl diphosphate (PM18) and DHAP (10:0) (PM21). These relationships suggested that the gut microbiota can influence host metabolism and the associated metabolic pathways furthermore.

## Discussion

Spleen deficiency is a common clinical symptom in TCM, which is a comprehensive pathological change of multiorgan and multisystem weakened functions, mainly due to reduced digestion and absorption function. Based on the TCM theory of “bitter cold injures the spleen”, the classical animal model of spleen deficiency caused by *Sennae Folium* was used in this experiment (Zhang et al., 2020b; Zhang et al., 2021). The blood routine, organ index, pathological sections and a series of physiological and biochemical indexes closely related to the model of spleen deficiency were selected to evaluate the traditional efficacy of JF in tonifying the spleen. The results showed that JF could improve the activity of amylase which was a key enzyme for digestion and absorption of sugars. The increase of its activity means that JF could enhance the metabolism and absorption of nutrients and improve the digestive function of spleen-deficiency rats (Haq et al., 2020). After the administration of JF, the level of gastrin was increased, which could stimulate the secretion of gastric acid, pepsin and trypsin (Zhao et al., 2011), promote the contraction of gastric antrum, increase the flow of gastric mucosa, and nourish the mucosa of gastrointestinal tract. The decrease of somatostatin content indicated that JF could reduce the inhibitory effect on gastric emptying and intestine peristalsis, enhance the secretion of gastric acid and pepsin, as well as the absorption of carbohydrates, amino acids and other nutrients in the small intestine (Rai et al., 2015; Schubert and Rehfeld, 2020). By reducing the white blood cell count and increasing lymphocyte ratio in the blood, JF were found to enhance immunity and relieve inflammation. JF also demonstrated intestinal protection by alleviating small intestinal tissue damage caused by spleen deficiency. Taken together, it could be hypothesized that JF can achieve the effect of nourishing the spleen by regulating digestive system, immune system and energy metabolism.

To determine the metabolic changes related to the spleen deficiency model in rats and the interventional role of JF, metabolomic studies were performed on their plasma and urine samples based on UHPLC-QTOF/MS technology. After multilevel statistical analysis, 31 potential biomarkers were identified, which were involved in D-glutamine and D-glutamate metabolism, retinol metabolism, pentose and glucuronide interconversion, alanine, aspartate and glutamate metabolism, nicotinate and niacinamide metabolism, ether lipid metabolism, selenoamino acid metabolism, glycolysis or gluconeogenesis. Differential metabolites and corresponding metabolic pathways seem to have diagnostic and prognostic functions in spleen deficiency, but these need to be further investigated. After intervention with JF, the retinol metabolism, pentose and glucuronate interconversions, nicotinate and niacinamide metabolism, ether lipid metabolism, and selenoamino acid metabolism were improved in spleen deficiency rats. The results showed that JF could nourish the spleen, and the possible mechanism of invigorating spleen could be presumed by metabolomics.

The potential biomarkers of retinyl palmitate (PM11), retinyl ester (PM15) and vitamin A (retinol, UN9) were all involved in the retinol metabolism. Vitamin A presented as different molecules forms, mainly including retinol, provitamin A carotenoids, and retinyl esters (Borel and Desmarchelier, 2017). Retinyl palmitate is also a retinyl ester, the main form of dietary prefabricated vitamin A (Ribaya-Mercado et al., 1994). Vitamin A is essential to human health and plays important roles in vision, growth, fertility and immune functions (Underwood, 1994). The metabolism of plasma retinyl palmitate, retinyl ester and urine vitamin A (retinol) in the rats of model group were disordered compared to those in the control group. It could be speculated that the spleen deficiency leads to the disorder of retinol metabolism, which may cause the impairment of vision, growth, fertility and immune function. Besides, it has been reported that the administration of taurocholic acid (PM14) could enhance vitamin A absorption in rats, and studies revealed that a complex correlation existed between vitamin A metabolites and bile acid (Saeed et al., 2017). The level of taurocholic acid in model rats’ plasma was down-regulated, which indirectly confirmed that the retinol metabolism has been disordered on the other hand.

Deoxycholic acid 3-glucuronide (PM6) is a metabolite of deoxycholic acid generated in the liver by UDP glucosyltransferase and involved in pentose and glucuronate interconversions. Deoxycholic acid and taurocholic acid (PM14) are both bile acids, used as detergents to dissolved fats for intestinal absorption, and then reabsorbed by themselves. Bile acids were physiological detergents that facilitate excretion, absorption, and transport of fats, sterols and other hydrophobic nutrients in the intestine and liver (Claudel et al., 2005). They stimulate bile flow and intestinal motility, regulate lipid secretion and glucose levels, which are essential for the solubilization and absorption of dietary lipids and fat-soluble vitamins (Keely et al., 2022; Mohammed et al., 2022). It has been implicated in the regulation of all the key enzymes for cholesterol homeostasis. At the same time, there are significant effects on gastrointestinal motility and secretory function, intestinal barrier permeability, apoptosis, glucose metabolism and inflammatory response regulation (Panek-Jeziorna and Mulak, 2017). In addition, studies have shown a complex interaction between bile acids and intestinal flora, while bile acids are also essential chemical communication components linking the intestinal flora and the intestinal immune system (Biagioli et al., 2021). Bile acids induce diarrhea by increasing colonic viability and secretion, and affect inflammation and microbiota (Camilleri and Vijayvargiya, 2020). The disorder of deoxycholic acid 3-glucuronide and taurocholic acid indicated the dysfunction of pentose and glucuronate inter-conversions (Zhang et al., 2012). Furthermore, it could be found that bile acids were involved in the pathological process of spleen deficiency and may be a key factor for digestion and absorption.

Niacinamide (UN3) is considered as one of the main participate in nicotinate and niacinamide metabolism and also an indispensable compound of coenzyme niacinamide adenine dinucleotide (NAD). Niacinamide participates in oxidation-reduction reactions, intervenes in energy metabolism and other physiological processes, plays an important role in lipid metabolism, carbohydrate anaerobic decomposition and tissue respiration hyper-oxidation processes (Mejía et al., 2018). When it participates in the NAD salvage pathway in mammals, the process needs adenosine triphosphate (ATP) to execute. The depletion of ATP affects NAD regeneration, and results in the accumulation of niacinamide (Skordi et al., 2007; Yu et al., 2017). In this study, increase of niacinamide in urine may be due to ATP depletion which is subsequently excreted in urine, which speculated that the metabolism of nicotinate and niacinamide in rats with spleen deficiency induced by *Sennae Folium* was interfered. In addition, nicotinate and niacinamide metabolism are involved in protein and sugar metabolism and are associated with inflammatory responses. Nicotinate has also been proved to alleviate intestinal inflammation and promote the repair of intestinal mucosal barrier injury (Li et al., 2017). After JF intervention, the content of niacinamide decreased, which was considered to be related to the improvement of diarrhea symptoms in spleen deficiency rats, the regulation of nicotinate and niacinamide metabolism, the alleviation of intestinal smooth muscle contraction and the decrease of energy demand.

After JF intervention, the relative contents of retinyl palmetate, retinyl ester, vitamin A, deoxycholic acid 3-glucuronide, taurocholic acid and niacinamide returned to the normal level. The results indicated that JF can improve spleen deficiency by altering retinol metabolism, pentose and glucuronate interconversions, nicotinate and niacinamide metabolism, so as to regulate digestive and absorption system, immune system and energy metabolism system. In addition, the metabolites with significant changes should be validated in subsequent experiments to confirm their roles.

Based on 16S rDNA sequencing technology, the structure of intestinal flora from phylum to genus was found to be altered in spleen-deficiency rats compared with the control group. The administration of JF significantly improved the elevated relative abundance of *Allobaculum*, *Turicibacter*, *Corynebacterium*, *Proteus*, *Aerococcus*, *Enterococcus*, *Epulopiscium*, *Morganella*, *Collinsella* and *Brevibacillus* caused by spleen deficiency. *Aerococcus* is a conditioned pathogen that can cause osteomyelitis, septic arthritis, meningitis, endocarditis, bacteremia and other infections (Sahu et al., 2021). It has been reported that *Aerococcus* could regulate the immune response through the production of pro-inflammatory cytokines (Curty et al., 2017). *Proteus* is highly abundant in inflammatory bowel disease and induces colitis in specific pathogen-free (SPF) mice (Srivastava et al., 2020). The relative abundance of *Aerococcus* and *Proteus* increased significantly in model group, indicating that the occurrence of intestinal inflammation caused by spleen deficiency, which is consistent with the results of pathological section. *Enterococcus* is a potential pathogen (Ponziani et al., 2018), and is associated with impaired intestinal permeability, which can induce a higher susceptibility to intestinal inflammation through the production of gelatinases that damage the epithelial barrier (Li et al., 2019). *Corynebacterium* is a clinically important bacterium, which can be regarded as a nosocomial infection pathogen related to sepsis, endocarditis, surgical wound infection, especially often causes infections in patients with low immune function (De Carvalho et al., 2021). *Morganella* was originally thought to be the cause of diarrheal disease, and it is now considered to be an important conditioned pathogen (Singh et al., 2015). *Turicibacter* is a common bacterium in the gastrointestinal tract and feces of humans and animals, but its ecological role and pathogenic potential remain unclear (Auchtung et al., 2016). It was evident that JF extract could improve and alleviate the symptoms of spleen deficiency rats by reducing the number of pathogenic microorganisms, such as *Corynebacterium*, *Proteus*, *Aerococcus* and *Morganella*.

The relationship between potential biomarkers and differential intestinal flora was evaluated by correlation analysis in this study. The results demonstrated that niacinamide (UN3), 17α-hydroxypregnenolone (UN8) and vitamin A (UN9) were the differential metabolites highly expressed in the model group. Niacinamide is considered to be a compound mainly involved in the metabolism of nicotinate and niacinamide, and plays an important role in lipid metabolism, anaerobic decomposition of carbohydrates, and hyperoxidation of tissue respiration (Mejía et al., 2018). 17α-Hydroxypregnenolone is the precursor of adrenal and gonadal steroid hormones, and often used as a sign of congenital adrenal hyperplasia and gonadal dysfunction (McKenna et al., 1977; Včeláková et al., 2007). Vitamin A participates in retinol metabolism and has a variety of physiological functions such as maintaining normal visual function, promoting cell growth and differentiation, regulating immune system function, and inhibiting tumor growth (Carazo et al., 2021). *Aerococcus*, as a conditioned pathogen, can regulate the immune response by production of pro-inflammatory cytokines, and its relative abundance is presumed to be elevated due to spleen deficiency diarrhea (Curty et al., 2017). According to Pearson’s correlation coefficient, *Aerococcus* was found to be positively correlated with niacinamide, 17α-hydroxypregnenolone, and vitamin A in urine, while JF was able to improve the elevated relative abundance of *Aerococcus* caused by spleen deficiency. It could be speculated that the elevated relative abundance of *Aerococcus* could further drive the pathological process of spleen deficiency by affecting retinol metabolism, nicotinate and niacinamide metabolism. Aerococcus may be a candidate strain for predicting and treating spleen deficiency and related disordered metabolic pathways.

In conclusion, this study combined plasma and urine metabolomics and 16S rDNA sequencing technology to explore the mechanism of JF in improving spleen deficiency rats, and to find the correlation of related metabolites and intestinal flora. The results suggested that JF may exert its spleen-supplementing effect by regulating retinol metabolism, pentose and glucuronate interconversions, nicotinate and niacinamide metabolism and intestinal flora diversity in spleen-deficiency rats. *Aerococcus* may be a candidate strain for predicting and treating spleen deficiency and related disordered metabolic pathways. Importantly, these findings highlight the potential importance of the gut microbiota on the spleen-supplementing activities of JF, presenting a new perspective on the mechanism of this herbal medicine, which would lay a foundation for further development of JF in clinical and daily health care application. Further work should focus on determining the most effective ingredients in JF for supplementing spleen. Moreover, fecal microbiome transplantation can be performed to analyze the key pathways triggered by JF.

## Data Availability Statement

The datasets presented in this study can be found in online repositories. The names of the repository/repositories and accession number(s) can be found below: https://www.ncbi.nlm.nih.gov/, PRJNA793571 https://www.ebi.ac.uk/metabolights/, MTBLS4257.

## Ethics Statement

The animal study was reviewed and approved by the Animal Ethics Committee of Nanjing University of Chinese Medicine.

## Author Contributions

YL, SG, and JD conceived, designed, and supervised the study. YL performed the experiments. YY, YL, and SG wrote the manuscript. YY, YL, and ES analyzed data. HY and XM revised the manuscript. WT, ES, YN, and DQ contributed reagents or analytical tools. All authors contributed to manuscript revision, read, and approved the submitted version.

## Funding

This work was supported by the National Natural Science Foundation of China (No. 81873189, 81473538), the Major Natural Science Research Project of Colleges and Universities in Jiangsu Province (18KJA360006), the Key R &D Program of Jiangsu Province (BE2021912), and National Key R&D Program of China (No. 2020YFC1712700).

## Conflict of Interest

The authors declare that the research was conducted in the absence of any commercial or financial relationships that could be construed as a potential conflict of interest.

## Publisher’s Note

All claims expressed in this article are solely those of the authors and do not necessarily represent those of their affiliated organizations, or those of the publisher, the editors and the reviewers. Any product that may be evaluated in this article, or claim that may be made by its manufacturer, is not guaranteed or endorsed by the publisher.
