# Supplementary material for: Elucidation of the Reinforcing Spleen Effect of Jujube Fruits Based on Metabolomics and Intestinal Flora Analysis
Source: Front Cell Infect Microbiol. 2022 Mar 24;12:847828. doi: 10.3389/fcimb.2022.847828 (PMC8987507; doi:10.3389/fcimb.2022.847828)
Supplement: Supplementary file 1 [file Table_1.docx]

Supplementary Material

# Supplementary Figures and Tables

**Table S1**  Potential biomarkers selected and identified between spleen deficiency model rats and normal rats

| **No.^a^** | **t_R_/min** | **m/z** | **Metabolite** | **Trend ^b^** | **Ion mode** | **HMDB** | **PubChem** | **KEGG** |
| --- | --- | --- | --- | --- | --- | --- | --- | --- |
| PM1 | 1.06 | 113.9659 | 1-pyrroline-2-carboxylic acid | ↓ | + | HMDB0006875 | 440046 | C03564 |
| PM2 | 1.08 | 169.0377 | Phosphoenolpyruvic acid | ↑ | + | HMDB0000263 | 1005 | C00074 |
| PM3 | 5.48 | 274.2736 | Heptanoylcarnitine | ↑ | + | HMDB0013238 | 6426896 | - |
| PM4 | 5.86 | 362.3272 | N-arachidonoyl glycine | ↑ | + | HMDB0005096 | 5283389 | - |
| PM5 | 7.35 | 568.3419 | LysoPC(22:6(4Z,7Z,10Z,13Z,16Z,19Z)) | ↑ | + | HMDB0010404 | 10415542 | C04230 |
| PM6 | 7.56 | 569.3457 | Deoxycholic acid 3-glucuronide | ↑ | + | HMDB0002596 | 53477755 | C03033 |
| PM7 | 8.27 | 184.0732 | Selenohomocysteine | ↑ | + | HMDB0004119 | 440763 | C05698 |
| PM8 | 9.83 | 510.356 | LysoPC(O-18:0) | ↑ | + | HMDB0011149 | 2733532 | C04317 |
| PM9 | 10.35 | 482.3254 | N-acetyl-leukotriene E4 | ↑ | + | HMDB0005084 | 53477792 | C11361 |
| PM10 | 10.36 | 341.307 | 9-hexadecenoylcholine | ↑ | + | HMDB0013208 | 22155839 | - |
| PM11 | 10.7 | 525.3734 | Retinyl palmitate | ↑ | + | HMDB0003648 | 5280531 | C02588 |
| PM12 | 12.93 | 663.4534 | DG(22:6(4Z,7Z,10Z,13Z,16Z,19Z)/18:3(6Z,9Z,12Z)/0:0) | ↑ | + | HMDB0007771 | 53478522 | - |
| PM13 | 3.39 | 429.1944 | Ketoprofen glucuronide | ↑ | - | HMDB0010334 | 131281 | C03033 |
| PM14 | 3.59 | 514.2836 | Taurocholic acid | ↓ | - | HMDB0000036 | 440567 | C05122 |
| PM15 | 10.44 | 301.2175 | Retinyl ester | ↓ | - | HMDB0003598 | 5460164 | C02075 |
| PM16 | 11.07 | 570.3602 | LysoPC(22:4(7Z,10Z,13Z,16Z)) | ↑ | - | HMDB0010401 | 52924039 | C04230 |
| PM17 | 11.08 | 510.094 | 3-methyl-1-hydroxybutyl-ThPP | ↑ | - | HMDB0006865 | 23724625 | C15974 |
| PM18 | 11.11 | 585.3525 | All-trans-hexaprenyl diphosphate | ↑ | - | HMDB0012188 | 5280413 | C01230 |
| PM19 | 12.81 | 579.3899 | Vitamin K2 | ↑ | - | HMDB0030017 | 5283547 | C00828 |
| PM20 | 13.03 | 355.1597 | 5-amino-6-(5'-phosphoribitylamino)uracil | ↑ | - | HMDB0003841 | 333 | C04454 |
| PM21 | 13.04 | 323.2213 | DHAP(10:0) | ↑ | - | HMDB0011675 | 53481023 | - |
| PM22 | 13.84 | 269.2487 | Estrone | ↑ | - | HMDB0000145 | 5870 | C00468 |
| UM1 | 1.05 | 143.1204 | 2-octenoic acid | ↓ | + | HMDB0000392 | 5282713 | - |
| UM2 | 1.79 | 142.089 | O-phosphoethanolamine | ↑ | + | HMDB0000224 | 1015 | C00346 |
| UM3 | 5.79 | 123.1159 | Niacinamide | ↑ | + | HMDB0001406 | 936 | C00153 |
| UM4 | 6.45 | 346.2235 | Eicosapentaenoyl ethanolamide | ↑ | + | HMDB0013649 | 5283450 | - |
| UM5 | 1.73 | 146.0824 | L-glutamic acid | ↑ | - | HMDB0000148 | 33032 | C00025 |
| UM6 | 2.44 | 276.0551 | S-(2-methylpropionyl)-dihydrolipoamide-E | ↓ | - | HMDB0006868 | 11953835 | C04424 |
| UM7 | 4.82 | 172.0974 | 2-oxoarginine | ↑ | - | HMDB0004225 | 558 | C03771 |
| UM8 | 9.8 | 331.1927 | 17α-hydroxypregnenolone | ↑ | - | HMDB0000363 | 91451 | C05138 |
| UM9 | 10.74 | 285.1847 | Vitamin A | ↑ | - | HMDB0000305 | 445354 | C00473 |

^a^ PM: metabolites from plasma; UM: metabolites from urine.

^b^ The trend is model group vs control group: ↑, increase; ↓, decrease.
